# Multi-objective optimization identifies cultivation strategies for balancing yield, quality, and resource efficiency in hydroponic netted melon

**DOI:** 10.1038/s41598-026-36171-4

**Published:** 2026-01-18

**Authors:** Mi Young Lim, Seungri Yoon, Se Jin Kim, Joo Hee Nam, Gyeong Lee Choi, Mi Young Roh, Hee Sung Hwang

**Affiliations:** 1 National Institute of Horticultural and Herbal Science, Rural Development Administration, Haman, 52054 South Korea; 2 Department of Agriculture, Forestry and Bioresources, Seoul National University, Seoul, 08826 South Korea; 3 IoT Research Center, Pusan National University, Busan, South Korea

**Keywords:** Trade-offs, Return on investment, Planting density, Economic value, Fruit quality, Hydroponics, Engineering, Plant sciences

## Abstract

Managing the trade-offs between yield maximization and fruit quality is a critical challenge in hydroponic melon production. To identify optimal cultivation strategies, we evaluated three netted melon cultivars (‘Dalgona’, ‘Hero’, and ‘Kingstar’) under restricted (10 L) versus standard (20 L) substrate volumes and two planting densities (3 vs. 4 plants/slab). We utilized Pareto frontier analysis to visualize the balance among fruit quality, resource use efficiency, and economic performance. The analysis revealed three distinct optimal strategies targeting specific market segments: profit-maximization (‘Hero’, 10 L, 4 plants/slab, 268% return on investment [ROI]), premium-quality focus (‘Dalgona’, 10 L, 3 plants/slab, 100% premium-grade), and resource-efficiency (‘Kingstar’, 10 L, 3 plants/slab, water productivity of 5.7 kg m⁻³). While increasing planting density enhanced total yield by 20.7%, it compromised internal fruit quality. The 10 L substrate volume achieved superior economic performance (ROI 42.9%–268.0%) across all cultivars by inducing a root restriction effect that improved sugar content and water productivity compared to the 20 L standard. These findings provide a quantitative decision-support framework enabling producers to select optimal parameter combinations tailored to their specific management objectives.

## Introduction

Controlled environmental agriculture (CEA) has emerged as an essential strategy worldwide to overcome the uncertainties associated with climate change and ensure food security^1^. There is an accelerated transition from soil-based to hydroponic cultivation systems, particularly to prevent soil-borne diseases and enable precise water and nutrient management^2–5^. This shift toward precision agriculture represents more than a simple change in cultivation methods; it is imperative to minimize input resources while maximizing productivity, break the vicious cycle between irrigated agriculture and climate change, and establish sustainable production systems^6–8^. Netted melon (*Cucumis melo* L.) is classified as a high-value crop with substantial profitability per unit area among protected horticultural crops^9–11^. However, successful hydroponic cultivation requires high economic efficiency to offset the initial facility investment costs and operating expenses for substrates and nutrient solutions^12,13^. Currently, growers face conflicting objectives of enhancing productivity and maintaining quality control; this highlights the urgent need to establish scientific and systematic cultivation strategies.

The profit structure of a hydroponic farm is determined by two key variables: yield and fruit quality. However, an inevitable trade-off exists between these factors, making it extremely difficult to identify the optimal balance points^14,15^. Generally, increasing planting density enhances total yield per unit area but triggers a “dilution effect,” where individual plant photosynthetic efficiency declines, which results in reduced fruit quality attributes such as sugar content^16–18^. Conversely, strict environmental controls or reduced planting density to produce high-quality fruits improves marketability but decreases total production, ultimately reducing farm income. Therefore, sustainable agricultural management requires multifaceted approaches that optimize economic value relative to input resources, and extend beyond simply maximizing biological yield.

Previous studies on the hydroponic cultivation of melons and cucurbits have predominantly focused on single-factor experiments that independently manipulated one variable (planting density^19–21^, substrate characteristics^22,23^, cultivation methods^24^, or water and nutrient management^25–28^) to verify its effects. Although these studies successfully demonstrated methods for maximizing the yield under specific conditions, they failed to comprehensively consider the interactions between closely linked production variables and resource efficiency. In particular, when using organic substrates such as coir, substrate volume represents a critical factor directly linked to initial investment costs. However, research quantitatively analyzing the economic balance between cost reduction and growth stability has been insufficient. Consequently, growers lack concrete guidelines for establishing customized strategies that align with their management objectives and resource constraints.

To overcome the limitations of previous research and simultaneously achieve conflicting multi-objective goals, this study introduces the Pareto frontier analysis, a methodology widely employed in engineering and economics^29,30^. Pareto optimization serves as a powerful tool for visually and quantitatively identifying optimal compromise points among yield, quality, and resource efficiency by deriving “non-dominated solutions,” which are states where no objective can be improved without sacrificing another^31^. Although multi-objective optimization studies aimed at enhancing resource use efficiency have recently been attempted in the long-term cultivation of crops, such as tomatoes and paprika^15,32–34^, cases applying this approach to derive optimal production strategies for short-term intensive crops, such as melons, remain rare.

The experimental design of this study aimed to ensure broad applicability of the results by selecting representative cultivars with distinctly differentiated genetic characteristics based on previous research databases rather than random variable settings. According to the morphological classification by Lim et al. (2020)^35^, ‘Kingstar’ was selected as a cultivar that is advantageous for large fruit production and exhibits vegetative-type characteristics with vigorous growth, large leaf area, and high fruit weight. In contrast, ‘Dalgona’ was selected as a model for high-quality small fruit production, displaying reproductive-type characteristics with somewhat weaker vigor and smaller fruit size, but superior sugar accumulation capacity and short internode length. Additionally, by including ‘Hero,’ which possesses intermediate characteristics between these two cultivars, we sought to comprehensively elucidate the physiological and economic responses of melon cultivars with diverse genetic backgrounds under controlled environmental conditions.

The substrate volume settings were also strategically designed to compare conventional cultivation practices with novel approaches aimed at maximizing resource efficiency. The 20 L substrate currently used as a standard in hydroponic farms is advantageous for securing water and nutrient buffering capacity in the root zone, thereby enhancing cultivation stability^25,36,37^, but may impose cost burdens. Meanwhile, research has reported that water limitation conditions alter crop water use efficiency (WUE) and induce physiological adaptation, affecting sugar accumulation in fruits^28,38,39^. Specifically, the 10 L substrate established as a control treatment aimed to reduce substrate costs and promote reproductive development and sugar accumulation by imposing appropriate water stress through root restriction. By establishing contrasting substrate conditions with 10 and 20 L substrate volumes, this study sought to clearly demonstrate the Pareto efficiency of resource constraints on melon sugar accumulation and yield performance.

Through this strategic experimental design, the present study focused on elucidating the mechanisms that maximize input-output efficiency in hydroponic melon production systems, beyond simple cultivar comparisons or substrate volume experiments. We will conduct an in-depth analysis of how restricted root zone environments (10 L) can physiologically compensate for light competition and fruit enlargement problems that may occur under high-density cultivation conditions, and ultimately how this affects farm profitability. This aligns with contemporary agricultural demands for producing high-quality produce while minimizing resource input. The results derived through the Pareto analysis can provide practical decision-making criteria for growers operating in diverse management environments.

In conclusion, the objective of this study was to quantify the trade-off relationships among yield, quality, and resource efficiency in hydroponic melon cultivation using Pareto frontier analysis and to present optimal cultivation guidelines aligned with growers’ management objectives. Specifically, we aimed to provide solutions for optimally combining cultivars, substrate volume, and planting density to meet diverse needs, including farms targeting profit maximization, premium-quality production, and eco-friendly and resource-conserving approaches. This approach is expected to enhance the competitiveness of the hydroponic melon industry and contribute to the establishment of precise production strategies for sustainable agriculture.

## Results

### Yield, water productivity, and fruit quality characteristics

The total fruit yield per unit area increased markedly with increasing planting density (*P*$\:\:\:<$ 001, Table 1). The ‘Hero’ cultivar achieved the highest yield of 4,339 kg/10a under the 10 L substrate volume with 4 plants/slab, and the ‘Kingstar’ cultivar also demonstrated a high total yield of 4,161 kg/10a under the same conditions. In contrast, the yield of ‘Dalgona’ was 3,466 kg/10a under the same 10 L, 4 plants/slab, showing 20.1% and 16.7% lower yields than ‘Hero’ and ‘Kingstar,’ respectively. Although increasing the planting density from 3 to 4 plants/slab significantly increased the total yield across all cultivars, the main effect of substrate volume on yield was not statistically significant (*P* $\:\:>$ 0.05).

**Table 1:** Total yield, water productivity (WP), fertilizer cost, and substrate cost of netted melon fruits grown under different combinations of cultivar (C), substrate volume (S), and planting density (D).

Cultivar	Substrate volume (L)	Planting density	Total yield (kg/10a)	WP^z^ (kg/m^3^)	Fertilizer cost (10a/USD)	Substrate cost (10a/USD)
‘Dalgona’	10	3	2712 c^y^	4.1 a	804.8	1085.4
		4	3466 a	3.3 c		1085.4
	20	3	2477 d	3.7 b		1392.9
		4	3141 b	3.1 d		1392.9
‘Hero’	10	3	3552 c	5.4 a	804.8	1085.4
		4	4339 a	4.2 c		1085.4
	20	3	3225 d	4.8 b		1392.9
		4	3846 b	3.8 d		1392.9
‘Kingstar’	10	3	3735 a	5.7 a	804.8	1085.4
		4	4161 a	4.0 c		1085.4
	20	3	3220 b	4.8 b		1392.9
		4	4101 a	4.1 c		1392.9
‘F-test’		C	***	***	–	–
		S	***	***		
		D	***	***		
		C×S	NS	NS		
		C×D	NS	***		
		S×D	NS	***		
		C×S×D	**	**		

^z^Water productivity (WP) was calculated as the total fruit yield (kg/10a) divided by the total amount of irrigation water supplied (m^3^/10a).

^y^Mean separation within columns by Tukey’s HSD test at *P* ≤ 0.05.

NS, *, **, ***: Not significant or significant at *P* < 0.05, 0.01, and 0.001, respectively.

However, the substrate volume had a significant impact on water productivity (WP). The 10 L substrate treatments consistently exhibited higher WP than the 20 L treatments across all cultivars. Specifically, ‘Kingstar’ recorded the highest WP of 5.7 kg/m^3^ in the 10 L substrate, whereas ‘Dalgona’ showed the lowest WP (3.1 kg/m^3^) in the 20 L substrate with 4 plants/slab. This suggests that reducing the substrate volume to 10 L improves water-use efficiency without compromising areal yield.

**Fig. 1:**
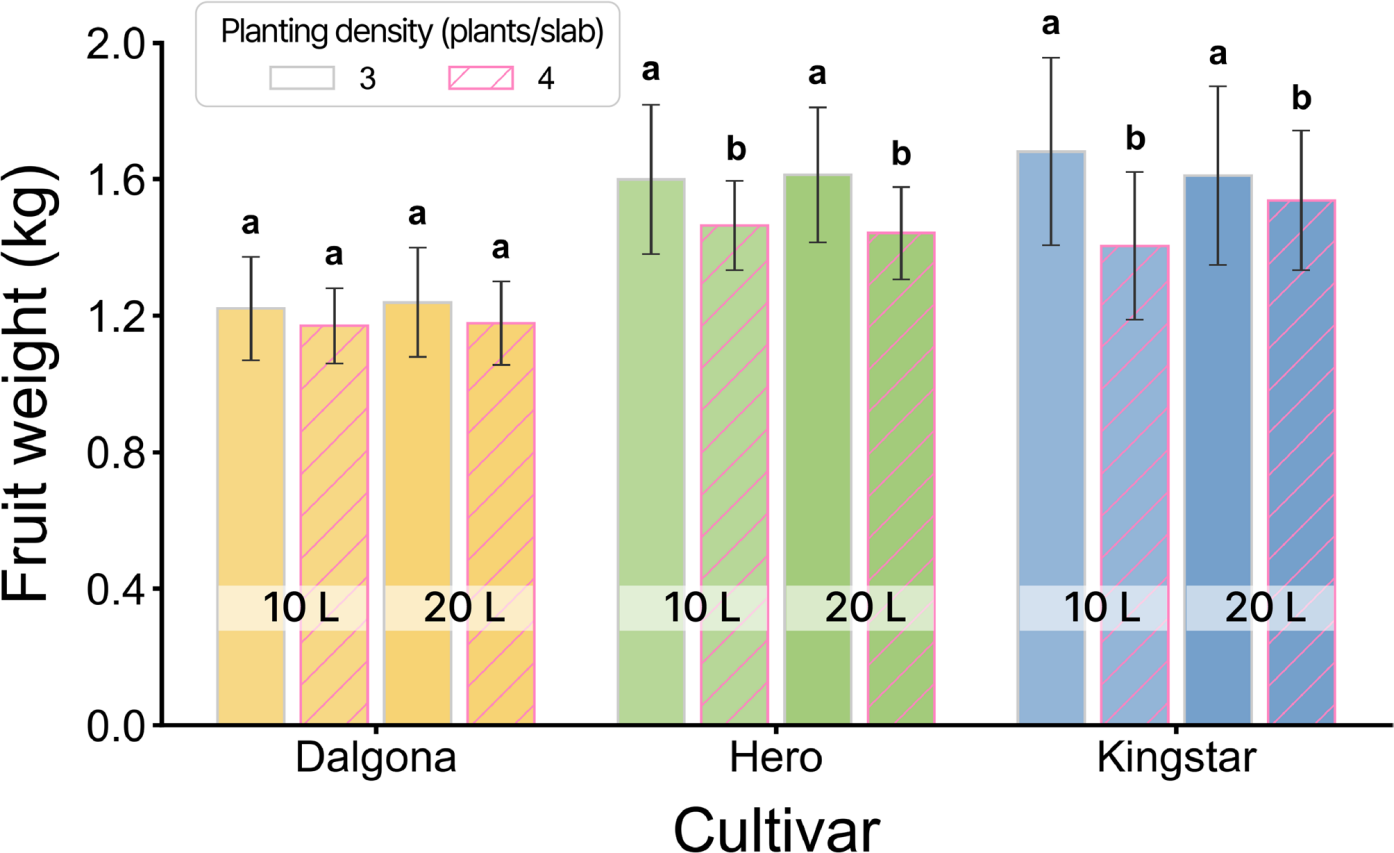
Effects of cultivar (‘Dalgona’, ‘Hero’, and ‘Kingstar’), substrate slab volume (10 L vs. 20 L), and planting density (3 vs. 4 plants/slab) on mean fruit weight (kg). Bars represent the mean values ± standard deviation. The color of the bars indicates the cultivar (yellow for ‘Dalgona’, green for ‘Hero’, blue for ‘Kingstar’), and the fill pattern distinguishes planting density (solid bars for 3 plants/slab; hatched bars for 4 plants/slab). Different lowercase letters above the bars indicate significant differences among treatments within each cultivar according to Tukey’s HSD test at *P* < 0.05. Three-way ANOVA results: Cultivar (C), *P* < 0.001; Slab volume (S), NS; Planting density (D), NS; *C* $\:\times\:$ *S*, NS; C $\:\times\:$ D, NS; S $\:\times\:$ D, NS; C $\:\times\:$ S $\:\times\:$ D, *P* < 0.01. (NS: not significant).

Regarding individual fruit weight, a significant three-way interaction (*P* $\:<$ 0.01) was observed among cultivar, substrate volume, and planting density (Fig. 1). Although fruit weight generally decreased as planting density increased, the magnitude of this reduction was cultivar dependent. ‘Kingstar’ was the most sensitive to density stress; its fruit weight significantly decreased by approximately 17% (from 1.68 kg to 1.40 kg) in the 10 L substrate when density increased from 3 to 4 plants/slab. Conversely, ‘Dalgona’ and ‘Hero’ showed no statistically significant reduction in fruit weight under the same conditions, indicating superior tolerance to high-density environments.

Fruit morphological traits, including length, diameter, and flesh thickness, also showed significant three-way interactions (*P* $\:<$ 0.001; Table 2). Fruit dimensions generally followed the trend of fruit weight, with ‘Kingstar’ being the largest and ‘Dalgona’ the smallest. The fruit shape index remained stable at approximately 1.0 (0.99–1.10) in all treatments, thereby indicating that the spherical shape was maintained regardless of cultivation conditions. ‘Hero’ exhibited the thickest flesh (maximum 43.0 mm), while ‘Dalgona’ had the thinnest.

**Table 2:** Morphological and quality traits of netted melon fruit grown under different combinations of cultivar (C), substrate volume (S), and planting density (D).

Cultivar (C)	Substrate volume (L) (S)	Plants/slab (D)	Fruit length (cm)	Fruit diameter (cm)	Fruit shape^z^	Net grade^y^	Flesh thickness (mm)
‘Dalgona’	10	3	13.7 a^x^	13.4 a	1.01 a	1.3 a	40.6 a
		4	13.1 d	13.1 c	0.99 c	1.2 a	39.6 a
	20	3	13.6 b	13.4 a	1.01 a	1.4 a	40.2 a
		4	13.3 c	13.2 b	1.00 b	1.5 a	35.3 b
‘Hero’	10	3	15.5 b	14.3 b	1.10 a	1.3 a	43.0 a
		4	14.9 d	13.8 c	1.09 a	1.2 a	40.1 cd
	20	3	16.0 a	14.4 a	1.07 a	1.4 a	41.0 b
		4	15.1 c	13.8 cd	1.09 a	1.4 a	40.3 c
‘Kingstar’	10	3	15.6 a	14.5 a	1.08 a	1.4 a	39.7 a
		4	14.6 b	14.0 b	1.03 c	1.6 a	39.3 a
	20	3	15.3 a	14.4 a	1.07 a	1.5 a	40.8 a
		4	15.0 a	14.3 a	1.05 b	1.5 a	40.0 a
‘F-test’		C	***	***	***	***	***
		S	***	***	NS	***	***
		D	***	***	***	NS	***
		C×S	***	NS	***	*	***
		C×D	***	***	***	*	***
		S×D	***	NS	***	NS	*
		C×S×D	***	***	NS	NS	***

z Expressed as the ratio of fruit length to fruit diameter. Values closer to 1 indicate a more spherical fruit shape.

y Net grade scale (based on RDA standards): 1 = excellent, 2 = good, 3 = average, 4 = poor, 5 = bad.

x Mean separation within columns by Tukey’s HSD test at *P* ≤ 0.05.

NS, *, **, ***: Not significant or significant at *P* < 0.05, 0.01, and 0.001, respectively.

Netting quality was significantly affected by the main effects of cultivar and substrate volume (*P* $\:<$ 0.001), whereas the effect of planting density was not significant. All cultivars produced fruit with excellent to good netting (net grade < 1.7). Notably, increasing the substrate volume from 10 to 20 L resulted in slightly higher net index scores (indicating rougher or poorer quality) across cultivars. For instance, ‘Dalgona’ showed a net index of 1.25 in the 10 L substrate volume compared to 1.45 in the 20 L substrate volume, which suggested that the root restriction effect of the 10 L volume may enhance net formation.

**Fig. 2:**
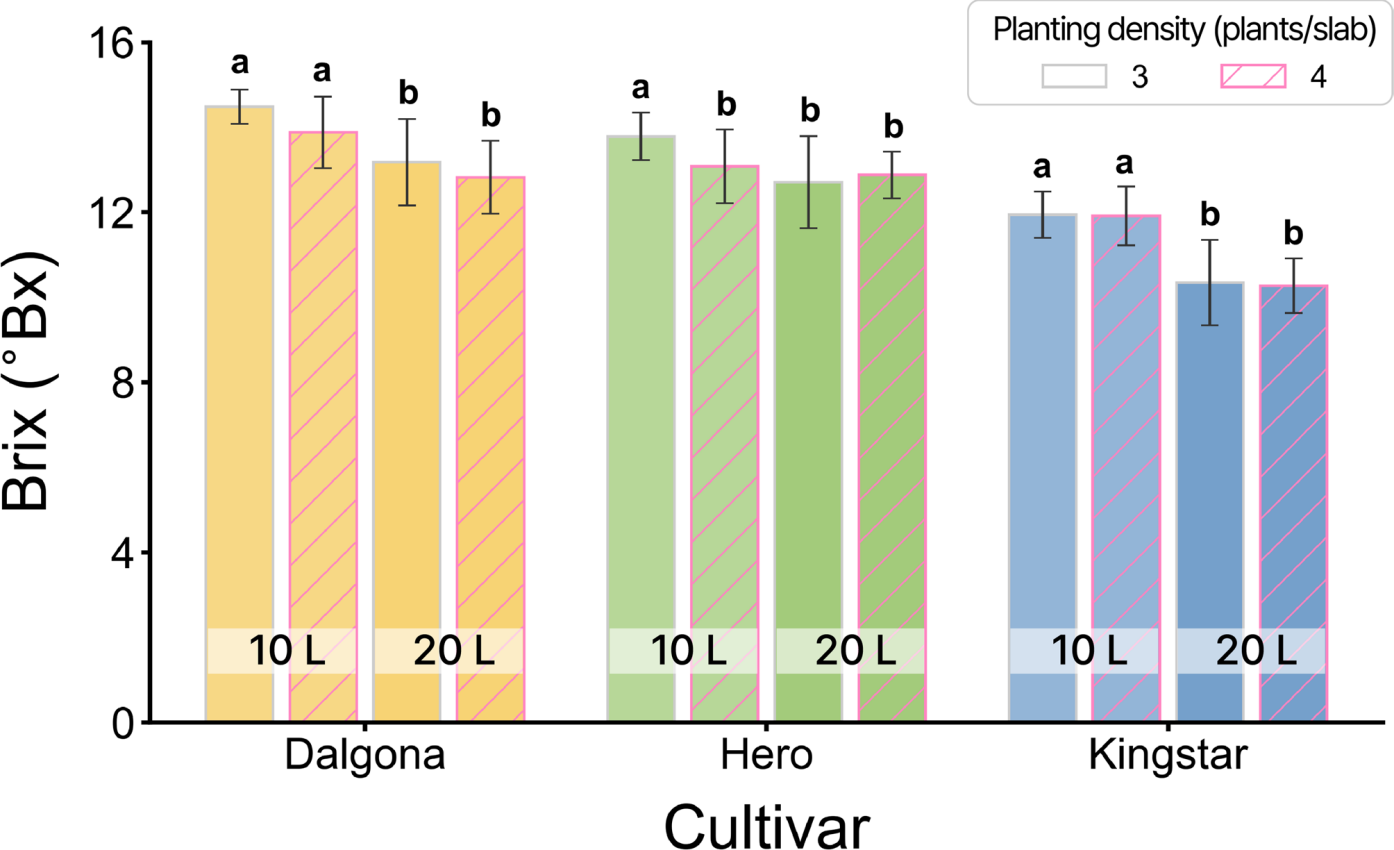
Effects of cultivar (‘Dalgona’, ‘Hero’, and ‘Kingstar’), substrate slab volume (10 L vs. 20 L), and planting density (3 vs. 4 plants/slab) on sugar content (Brix) of melon fruits. Bars represent the mean values ± standard deviation. Bars are colored by cultivar (yellow: ‘Dalgona’, green: ‘Hero’, blue: ‘Kingstar’), and the fill pattern distinguishes planting density (solid: 3 plants/slab; hatched: 4 plants/slab). Different lowercase letters above the bars indicate significant differences among treatments within each cultivar according to Tukey’s HSD test at *P* < 0.05. Three-way ANOVA results: Cultivar (C), *P* < 0.001; Slab volume (S), *P* < 0.001; Planting density (D), NS; C $\:\times\:$ S, NS; C $\:\times\:$ D, NS; S $\:\times\:$ D, NS; C $\:\times\:$ S $\:\times\:$ D, NS. (NS: not significant).

The sugar content (Brix) was significantly influenced by the main effects of cultivar and substrate volume (*P* $\:<$ 0.001), whereas the main effects of planting density and interaction effects were not significant (Fig. 2). This highlights that substrate volume is a critical determinant of sugar accumulation. Across all cultivars, the 10 L substrate volume consistently resulted in higher Brix values than the 20 L substrate volume. ‘Dalgona’ achieved the highest quality peaking at 14.5 °Bx in the 10 L, 3 plants/slab. In contrast, ‘Kingstar’ recorded the lowest Brix, dropping below 10.3 °Bx in the 20 L substrate volume. Consequently, restricted substrate volume proved to be more effective than density manipulation for improving internal fruit quality.

**Fig. 3:**
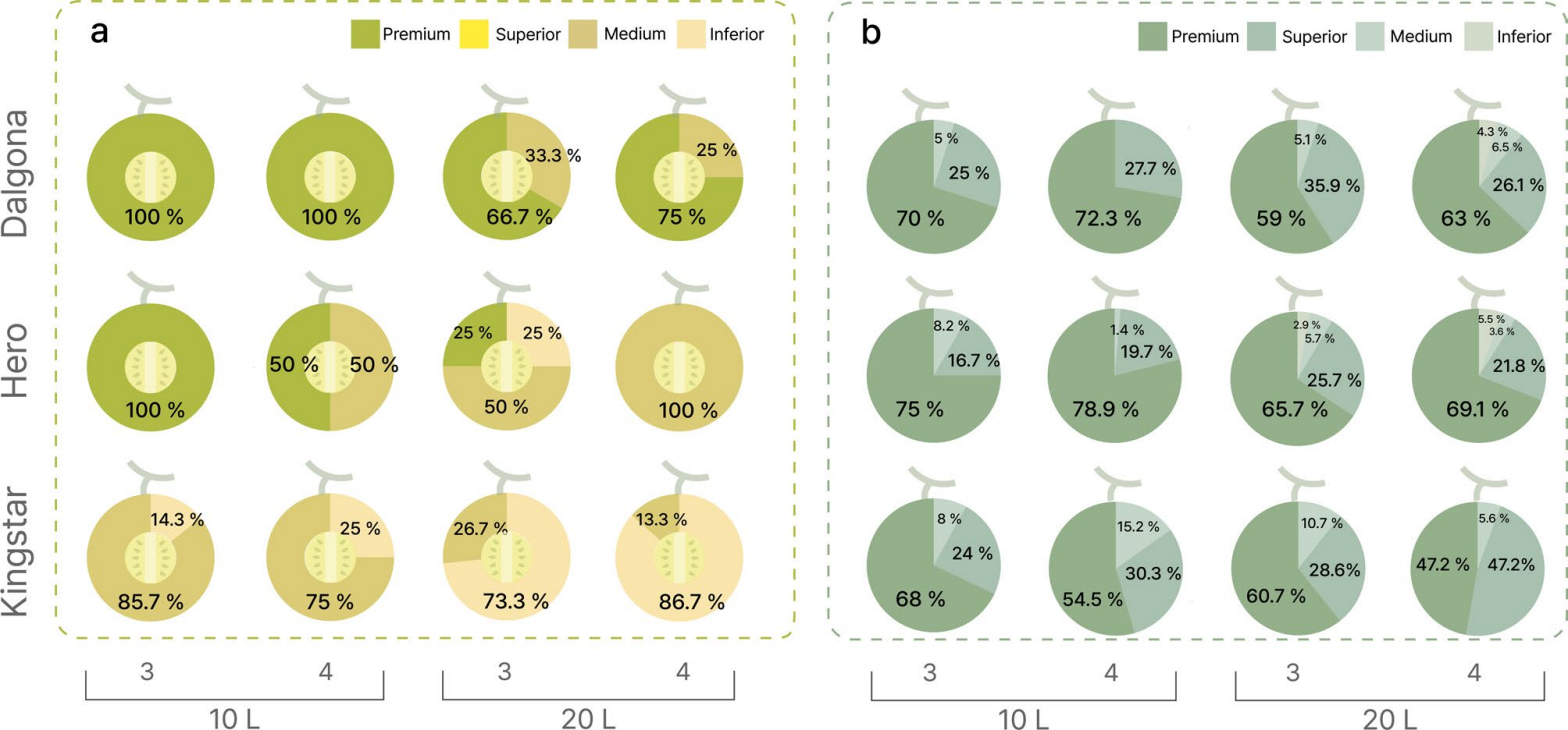
Market grade distribution of netted melon fruits based on quality assessment criteria. (**a**) Distribution based on sugar content analysis (°Bx) showing the percentage of fruits classified into four grades: Premium (dark green, ≥ 14°Bx), Superior (medium green, 12–14°Bx), Medium (yellow-green, 10–12°Bx), and Inferior (light brown, < 10°Bx). (**b**) Distribution based on net pattern analysis showing the percentage of fruits classified according to net density and uniformity: Premium (dark green), Superior (medium green), Medium (light green), and Inferior (lightest green). Each pie chart represents the grade composition for individual treatment combinations, with cultivars arranged vertically (‘Dalgona’, ‘Hero’, ‘Kingstar’) and substrate volume (10 and 20 L) and planting density (3 and 4 plants/slab) arranged horizontally. Percentages indicate the proportion of fruits in each grade category within each treatment, *n* = 500.

Market-grade distribution analysis revealed distinct quality differences among the cultivars based on sugar content (Fig. 3a). All fruits of the ‘Dalgona’ cultivar from the 10 L substrate volume were classified as premium grade (≥ 14 °Bx). Although the premium ratio decreased slightly for the 20 L substrate volume, it remained above 66%. The ‘Hero’ cultivar achieved 100% premium grade only in the 10 L substrate volume with 3 plants/slab, with the portion of medium grade fruits increasing as planting density and substrate volume increased.

The ‘Kingstar’ cultivar showed relatively poor performance, with premium ratios of only 13.3–26.7% across all experimental conditions, and medium and inferior grades accounting for 73.3–86.7%. The net grade-based evaluation showed a different pattern from sugar content, with all cultivars maintaining a premium and superior grade ratio above 85% regardless of substrate volume or planting density, thereby emphasizing uniformity in appearance (Fig. 3b).

### Comprehensive performance analysis

**Fig. 4:**
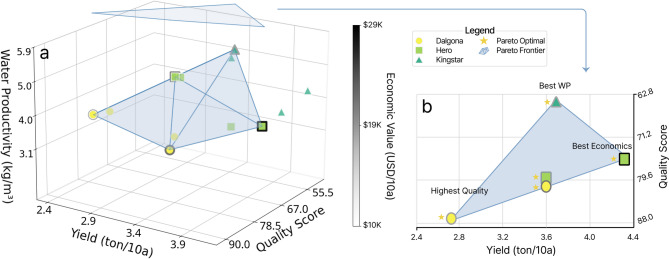
Three-dimensional Pareto frontier analysis demonstrating multi-objective trade-offs among quality, yield, and water productivity in hydroponic netted melon cultivation. (**a**) 3D scatter plot showing quality score (x-axis), yield (y-axis, ton/10a), and water productivity (z-axis, kg/m³) for 12 treatment combinations across three cultivars: Dalgona (○, yellow), Hero (□, green), and Kingstar (△, blue). Points are grayscale shaded by economic value ranging from $10K to $29K. The blue wireframe represents the Pareto frontier identifying non-dominated solutions, with gold-outlined points indicating Pareto-optimal treatments. (**b**) 2D projection of yield-quality trade-off with key optimal solutions labeled: “Highest Quality” (Dalgona 10 L, 3plants/slab), “Best Economics” (Hero 10 L, 4plants/slab), and “Best WP” (Kingstar 10 L, 3plants/slab). The blue shaded area represents the feasible solution space, demonstrating that no single treatment dominates across all objectives.

Multidimensional performance analysis enabled the identification of cultivation strategies optimized for different market segments and production objectives (Fig. 4a). A three-dimensional analysis integrating yield, quality score, and economic value revealed distinct differences among cultivar-treatment combinations, with each cultivar following unique optimization pathways. The greatest range in quality scores was found in the ‘Dalgona’ cultivar (70.62–87.67 points), although it showed the lowest total yield, at 2,477–3,466 kg/10a. The ‘Hero’ cultivar demonstrated intermediate performance, with quality scores of 70.35–81.55 points and yields of 3,225–4,339 kg/10a. In contrast, ‘Kingstar’ showed the highest irrigation water productivity, at 4.0–5.7 kg/m³, but the lowest quality scores, at 46.91–64.16 points. Pareto frontier analysis identified one–two optimal combinations for each cultivar, thus, confirming clear tradeoffs between yield, quality, and resource efficiency (Fig. 4b).

**Fig. 5:**
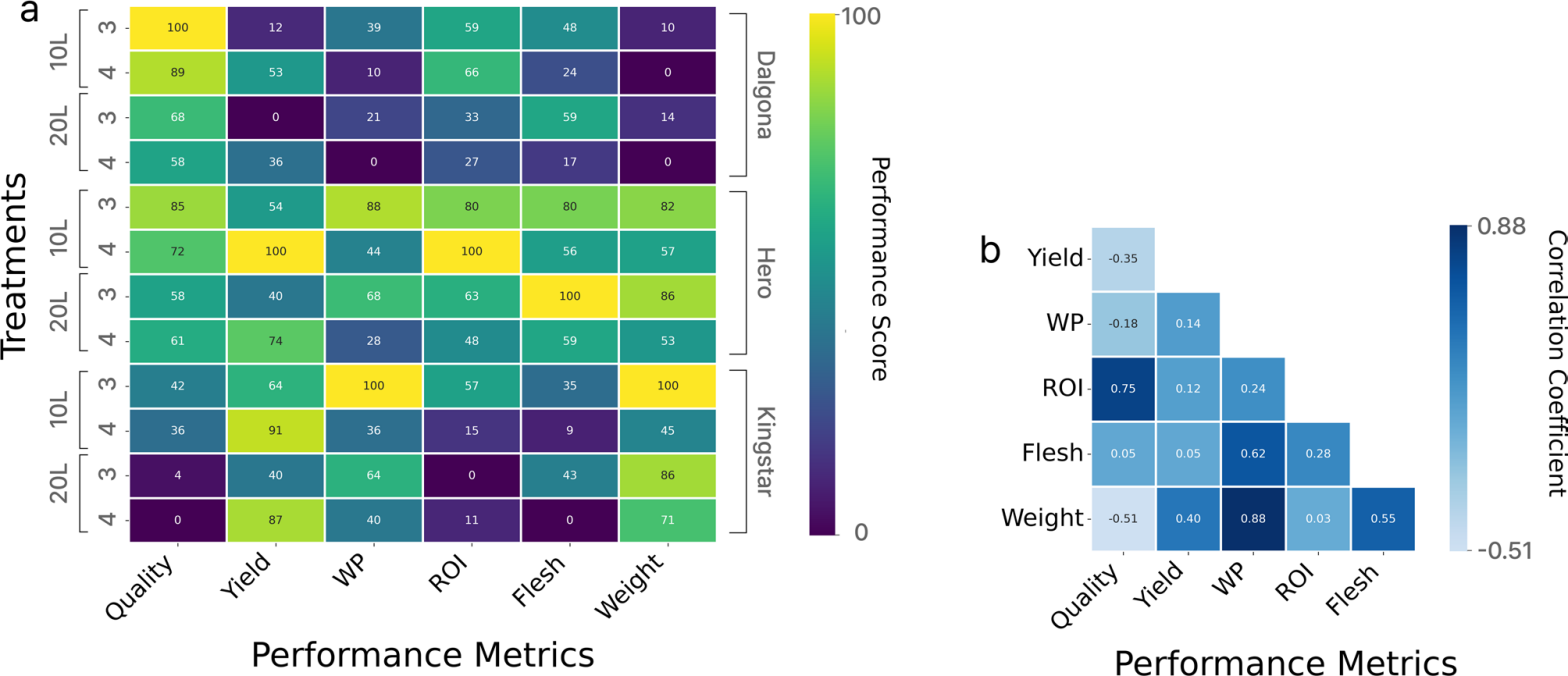
Comprehensive performance analysis of netted melon cultivation systems. (**a**) Treatment performance matrix showing normalized performance scores (0–100) across six key metrics for 12 cultivar-treatment combinations. Color intensity represents relative performance within each metric; yellow indicates highest performance (100) and dark purple lowest (0). Quality, quality score; Yield, total yield; WP, water productivity; ROI, return on investment as economic value; Flesh, mean flesh thickness; Weight, mean fruit weight. (**b**) Correlation matrix among performance metrics showing Pearson correlation coefficients. Blue shades indicate positive correlations, with darker blue representing stronger correlations (*r* approaching + 1.0).

Normalization of the six key performance indicators for the 12 treatment combinations on a scale of 0–100 revealed distinct performance patterns for each cultivar (Fig. 5a). In ‘Dalgona,’ the combination of 10 L substrate volume and 3 plants/slab achieved the highest quality score (100 points) but showed low yield (12 points) and economic value (39 points). The ‘Hero’ melons grown in 10 L substrate volume with 4 plants/slab scored 100 points in both yield and economic performance, demonstrating balanced high performance, with a quality score of 72 points at the upper-middle level. The ‘Kingstar’ cultivar excelled in irrigation water productivity (100 points) in the 10 L substrate volume with 3 plants/slab and achieved the highest yield (91 points) in the 10 L substrate volume with 4 plants/slab. In all 20 L substrate volume treatments, the quality scores and economic value were lower than those in the 10 L substrate volume treatments for the same cultivar.

Differences in performance according to substrate volume varied by cultivar. In the ‘Dalgona’ cultivar, quality scores decreased by an average of 10.4 points (10 L: 85.36 points → 20 L: 72.60 points) and economic value decreased by 55.8% as substrate volume increased. The ‘Hero’ cultivar showed decreases of 7.7 points and 22.7% in quality score and economic performance, respectively, whereas ‘Kingstar’ exhibited the largest quality score reduction of 13.7 points. Increased planting density (3 → 4 plants/slab) was accompanied by the contrasting effects of increased yield and quality deterioration. In the ‘Dalgona’ cultivar, the yield increased by an average of 20.7%, but the quality scores declined by 5.3 points. Similar trends were observed in the ‘Hero’ and ‘Kingstar’ melons, with quality scores decreasing by 4.1 and 3.3 points, respectively, indicating relatively smaller quality losses.

Correlation analysis among the performance indicators revealed that economic value showed a strong positive correlation with quality score (*r* = 0.75), whereas it exhibited a weak correlation with total yield (*r* = 0.12) (Fig. 5b). Irrigation water productivity showed a weak correlation with total yield (*r* = 0.14), but a high correlation with fruit weight (*r* = 0.88). Overall comprehensive performance analysis identified ‘Hero’ grown in the 10 L substrate volume with 4 plants/slab as the optimal combination for economic advantage (economic value ROI 268.0%/10a, yield 4,339 kg/10a). The ‘Dalgona’ melons in the 10 L substrate volume with 3 plants/slab were shown to be quality-first (quality score 87.67 points, economic value ROI 175.2%/10a), and the ‘Kingstar’ melons in the 10 L substrate volume with 3 plants/slab were the most resource-efficient (irrigation water productivity 5.7 kg/m³, flesh thickness 17.8 mm). These results suggest that no single optimal solution exists; rather, the cultivation strategy should match the target performance indicators set by producers.

### Economic analysis and cost efficiency evaluation

**Fig. 6:**
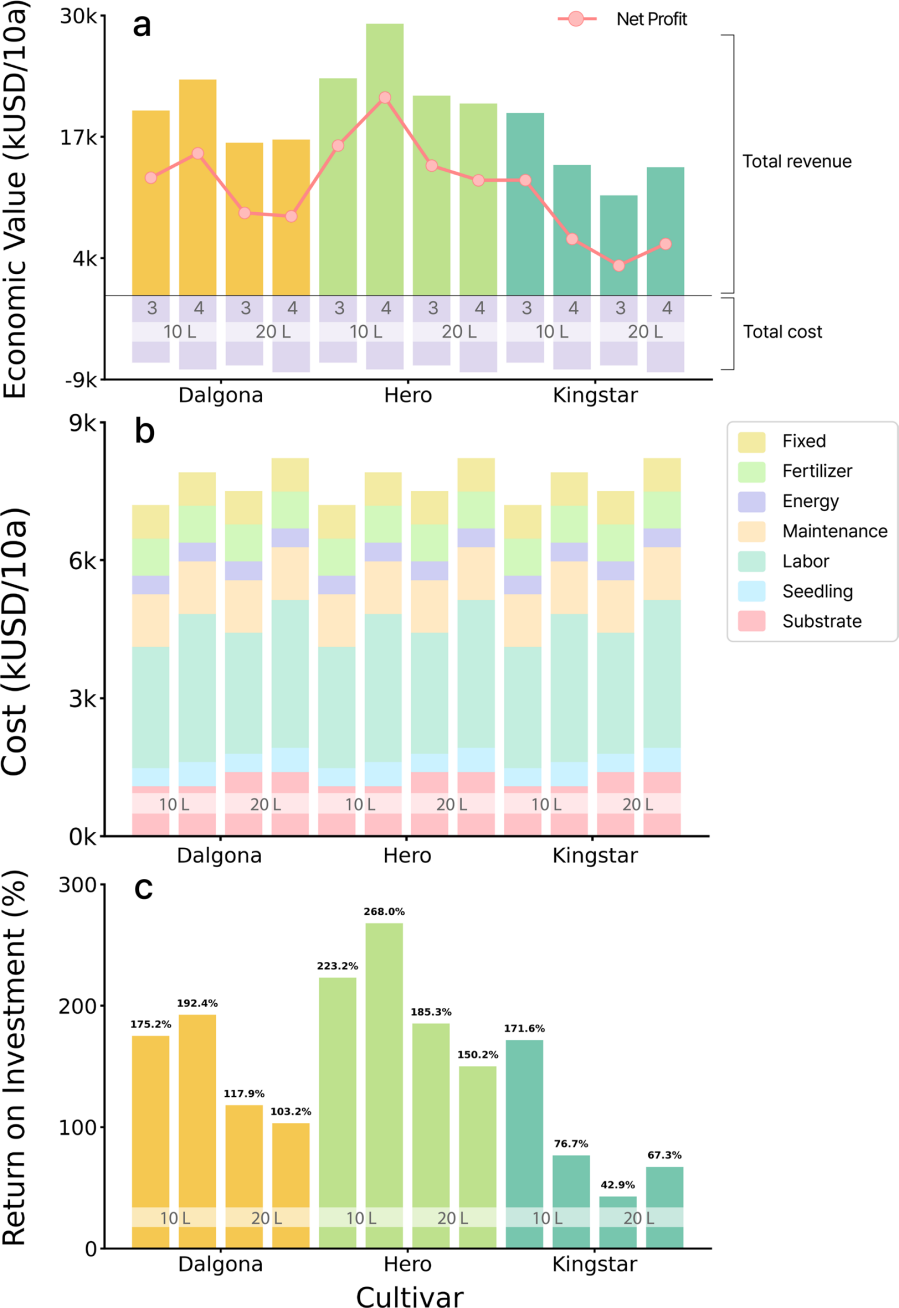
Economic profitability analysis of netted melon cultivars under different hydroponic cultivation systems. (**a**) Comparative analysis of total revenue, total cost, and net profit across three cultivars (‘Dalgona’, ‘Hero’, and ‘Kingstar’) under combinations of substrate volume (10 and 20 L) and planting density (3 and 4 plants/slab). Positive bars represent total revenue (colored by cultivar), negative bars represent total cost (light purple), and the red line with markers indicates net profit. The black horizontal line represents the break-even point. (**b**) Detailed cost structure breakdown showing the composition of production costs per treatment. Stacked bars illustrate seven major cost components: substrate (pink), seedlings (light blue), labor (mint green), maintenance (peach), energy (light purple), fertilizer (light green), and fixed costs (khaki). (**c**) Return on investment (ROI) analysis expressed as percentage values. Bars are colored according to cultivar identity, with numerical ROI values displayed above each bar. The horizontal black line at 0% indicates the profitability threshold. Cost calculations were based on actual agricultural survey data.

Economic analysis applying a quality-based differential pricing system revealed distinct profitability differences among the cultivars (Fig. 6a). The ‘Hero’ cultivar was found to have the best overall economic performance, achieving a maximum net profit of $21,213/10a and total revenue of $29,127/10a under 10 L substrate volume with 4 plants/slab. The 10 L substrate volume with three plants/slab also showed good profitability, with a total revenue of $23,262/10a. With the ‘Dalgona’ cultivar, net profit was estimated at $15,228/10a in the 10 L substrate volume with 4 plants/slab but decreased significantly, to $8,489–8,853/10a, when the melons were grown in the 20 L substrate volume. As for the ‘Kingstar’ melons, a net profit of $12,354/10a would be obtained with the 10 L substrate volumes with 3 plants/slab condition; however, profit is expected to decline sharply to $3,217–5,534/10a when the 20 L substrate volume is used. The results showed the highest sensitivity to the substrate volume.

When a standardized production cost structure was applied to each cultivation system, the difference in the total cost among the three systems was as high as 14.2%. For the same cultivar, the production cost was the lowest in the 10 L substrate volume with three plants/slab (averaging $7,198/10a) and highest in the 20 L substrate volume with 4 plants/slab (averaging $8,222/10a) (Fig. 6b). The increase in substrate volume (10 → 20 L) increased substrate costs by 28.3%, and the increase in planting density (3 → 4 plants/slab) increased seedling costs by 33.3%, which served as major cost escalation factors.

Return on Investment (ROI) was found to be highest with the ‘Hero’ cultivar grown in the 10 L substrate volume with 4 plants/slab, at 268.0% (Fig. 6c). ‘Hero’ melons grown in the less dense slabs (10 L, 3 plants) also resulted in a high ROI, at 223.2%. Dalgona yielded a good ROI of 175.2%–192.4% in the 10 L substrate volume, but this value decreased to 103.2%–117.9% in the 20 L substrate volume. Of the three cultivars, ‘Kingstar’ showed the lowest ROI under the same cultivation systems; ROI of 171.6% from ‘Kingstar’ melons grown in the 10 L substrate volume with 3 plants/slab declined sharply to 42.9–67.3% in the larger substrate volume (20 L). In summary, all three cultivation systems delivered positive ROI values, securing economic viability; however, the highest ROI (above 200%) was achieved with ‘Hero’ melons grown in the 10 L substrate volume.

## Discussion

The contrasting results of yield increase and quality deterioration with increasing planting density can be explained by the physiological principles of resource competition among plants and assimilation partitioning. Consistent with Lim et al. (2020)^19^, who reported a yield increase of approximately 20–33% with higher density, our study observed a 20.7% increase in total yield when planting density was increased from 3 to 4 plants/slab; however, this yield gain was accompanied by a 5–17% reduction in individual fruit weight. This reduction is attributed to intensified light competition within the canopy, which reduces photosynthetic efficiency and limits carbohydrate accumulation in the fruits during maturation^41–44^. Our study revealed cultivar-specific sensitivity to this density stress; the large-fruited cultivar ‘Kingstar’ showed greater relative reduction in fruit weight compared to the small-fruited ‘Dalgona.’ This suggests that cultivars with higher sink strength requirements for fruit enlargement are more susceptible to limitations under high-density conditions^45–48^.

Contrary to previous assumptions^49,50^, our rigorous factorial analysis revealed that substrate volume is a critical determinant of fruit quality and water productivity. While the total yield was not significantly compromised by reducing the substrate volume from 20 L to 10 L, the 10 L substrate volume significantly enhanced the internal fruit quality, particularly sugar content (Brix). This can be attributed to the root restriction effect, where limited root volume imposes mild water stress, thereby inhibiting excessive vegetative growth and shifting the physiological balance of the plant toward reproductive growth^51,52^. This mechanism aligns with research showing that regulated deficit irrigation or root restriction improves fruit quality traits, such as sugar accumulation and firmness, by concentrating assimilates in the fruit sink^54–56^. Furthermore, the significantly higher WP observed in the 10 L substrate volume (e.g., 5.7 kg/m³ for ‘Kingstar’) indicates that optimizing substrate volume can maximize resource use efficiency without sacrificing marketable yield.

The positive ROI (42.9–268.0%) across all cultivation systems provides important evidence of the economic viability of hydroponic netted melon production. Considering that the average income rate of commercial greenhouse melon farms is 45% in 2023^11^, the results demonstrate competitive economic performance for the hydroponic cultivation systems tested. Notably, the superior economic performance of the 10 L substrate volume resulted from complex synergies between reduced material costs and quality-driven price premiums. The weak correlation between water productivity and economic value (*r* = 0.24) indicates that water use efficiency alone cannot guarantee profitability, which is attributed to the low cost of agricultural water^57,58^. Conversely, the strong positive correlation between the quality score and economic value (*r* = 0.75) indicates that high-quality production is key to profitability enhancement.

**Fig. 7:**
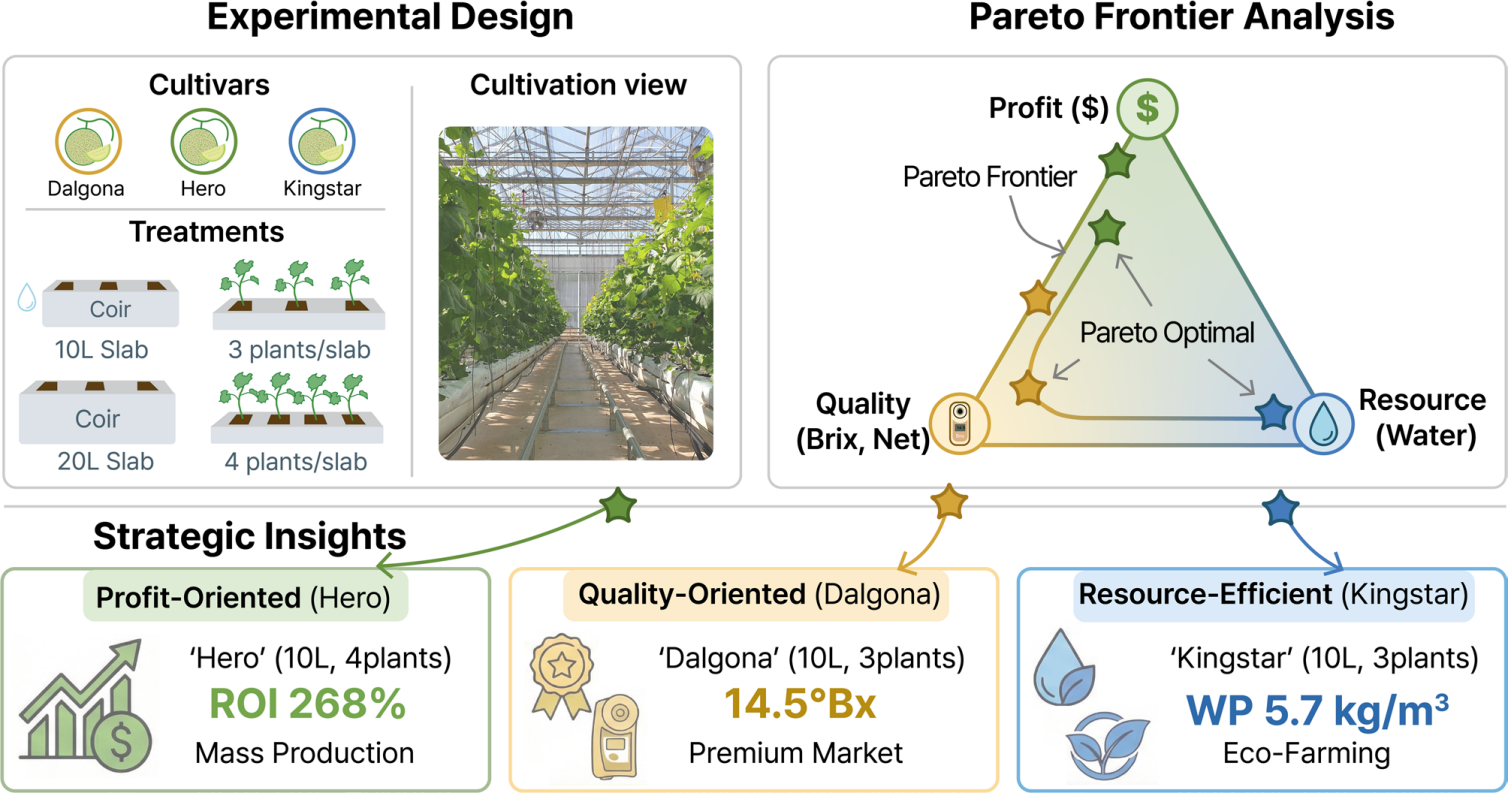
Schematic representation of the multi-objective optimization framework and strategic cultivation guidelines derived from this study. The upper panels illustrate the experimental design involving three cultivars and varying substrate volumes and planting densities. The top right panel depicts the Pareto frontier analysis used to evaluate trade-offs among profitability ($), fruit quality (Brix), and resource efficiency (water productivity). The bottom panel summarizes the three identified optimal strategies: Profit-Oriented (‘Hero’, 10 L, 4 plants/slab), Quality-Oriented (‘Dalgona’, 10 L, 3 plants/slab), and Resource-Efficient (‘Kingstar’, 10 L, 3 plants/slab), providing actionable criteria for precision management.

Pareto frontier analysis effectively visualizes trade-offs among conflicting objectives, thereby offering scientific evidence for strategic decision making (Fig. 7). The absence of a single optimal solution strongly suggests the need for producer-customized approaches: (1) Profit-Maximization (‘Hero,’ 10 L, 4 plants/slab) for mass markets, (2) Quality-Focus (‘Dalgona,’ 10 L, 3 plants/slab) for premium markets, and (3) Resource-Efficiency (‘Kingstar,’ 10 L, 3 plants/slab) for sustainable eco-farming. These distinct clusters provide actionable guidelines for growers to select the optimal parameter combinations based on management goals and market targets.

The limitations of this study include the use of standardized cost data and a single-year experimental period, which may not fully capture regional climatic variations or fluctuating market prices. Future research should focus on longitudinal studies across diverse environments to validate these optimization models. In addition, integrating real-time environmental control data with fruit growth modeling could further refine these strategies for smart farming applications.

## Conclusion

In this study, we quantified the complex trade-offs between yield, fruit quality, and resource efficiency in hydroponic netted melon production using a multi-objective optimization framework. Our findings demonstrate that substrate volume restriction (10 L) is a more effective strategy than planting density manipulation for enhancing internal fruit quality and water productivity, likely because of the physiological benefits of mild root restriction. Through Pareto frontier analysis, we identified three distinct optimal strategies: a profit-oriented strategy using ‘Hero’ (10 L, 4 plants/slab) for maximizing ROI; a quality-oriented strategy using ‘Dalgona’ (10 L, 3 plants/slab) for the premium market; and a resource-efficient strategy using ‘Kingstar’ (10 L, 3 plants/slab) for sustainable water use. These results challenge the conventional practice of using larger substrate volumes (20 L) and provide a scientific basis for adopting resource-saving and high-efficiency cultivation protocols. We suggest that future hydroponic systems prioritize cultivar-specific parameter optimization to achieve both economic and environmental sustainability.

## Materials and methods

### Cultivation and plant materials

This study was conducted in a venlo-type glass greenhouse (672 m²) at the Protected Horticulture Research Institute of the National Institute of Horticultural and Herbal Science, Haman (35.2°N, 128.4°E), Republic of Korea. Three muskmelon cultivars (*Cucumis melo* L.) were used: ‘Earl’s Kingstar’ (Nongwoobio Co., Ltd., Korea), ‘Hero’ (Farm Hannong Co., Ltd., Korea), and ‘PMR Dalgona’ (Lucky Seeds). Seeds were sown on January 29, 2020, in 50-cell trays filled with commercial nursery substrate (Tosil, Shinan Growth Co., Ltd., Korea) and grown for 36 days in a nursery room. The nursery room environment was maintained at daytime temperatures of 28–32 °C and nighttime temperatures of 18–20 °C until establishment, then gradually reduced to 14–16 °C after the first true leaf development to produce robust seedlings.

Transplantation into the greenhouse took place on March 5 when the plants had developed two to three true leaves. After transplantation, the plants were trained vertically using guide strings until pinching, and arranged in a zigzag pattern with adjacent plants. Lateral shoots below the 10th node were removed, whereas those from the 11th node onward were left intact. When female flowers appeared at nodes 11–13, pollinating bees were introduced into the greenhouse for approximately 10 days, and the fruits were set on the lateral shoots arising from nodes 11–13. Subsequently, one well-shaped fruit was retained and trained, whereas the rest were removed.

Plants were pinched above the 23rd node on March 31, after which the vegetative growth was monitored. Fruits were harvested on May 24, and their characteristics were evaluated. Pest and disease management was performed according to the standard integrated pest management (IPM) practices for hydroponic melon cultivation^59^. Cultural control measures included the disinfection of equipment and seeds before cultivation. Physical control involved the installation of sticky traps at the plant apex to control the whiteflies. Conventional pesticide applications were performed at 7–10 day intervals from 4 to 5 pm, diluted to the recommended concentration according to the crop protection product guidelines, and applied uniformly using a sprayer of more than 30 ml per plant to ensure thorough coverage of all cultivated crops.

**Fig. 8:**
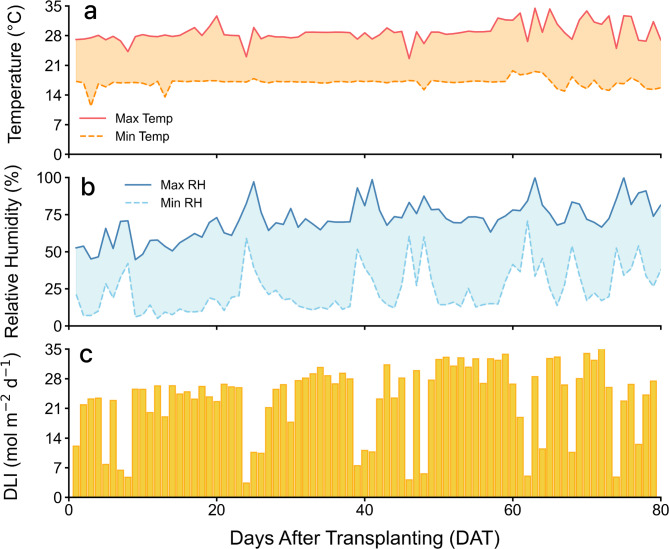
Environmental conditions during the cultivation period. (**a**) Daily maximum and minimum greenhouse temperatures (°C), (**b**) daily maximum and minimum relative humidity (%), and (**c**) daily light integral (DLI, mol·m⁻²·d⁻¹) recorded from transplanting to harvest. The x-axis represents days after transplanting (DAT).

During the cultivation period, the greenhouse temperature was maintained at a maximum/minimum of 28–30 °C/15–16 °C, relative humidity at 70–80%/20–30%, and the daily light integral (DLI) ranged from a maximum/minimum of 35.4/3.4 mol·m^−2^·d^−1^ (Fig. 8). Temperature, humidity, and light intensity were recorded using a data logger (CR1000; Campbell Scientific, Inc., Logan, UT, USA).

The growing medium specifications consisted of coir slabs (Daeyoung GS, Daegu, Korea) with a 50:50 ratio of chips to dust in two sizes: 90 × 15 × 7.5 cm (10 L) and 100 × 20 × 15 cm (20 L). Yamazaki solution, specifically formulated for melons, was used as the nutrient solution^60^. The nutrient solution electrical conductivity (EC) was maintained at 1.8–2.1 dS·m⁻¹ (pH 6.0) during the initial planting period and after fruit set. The irrigation volume was adjusted to achieve drainage rates of 20%, 30%, 40%, and 10% during the initial planting, flowering, fruit enlargement, and ripening stages, respectively. The drainage volume from both the substrate capacity and planting density treatments was continuously monitored to ensure similar irrigation volumes per unit area across treatments. To measure the irrigation volume, additional drip pins for nutrient solution collection were installed in the irrigation lines and daily irrigation data were collected in beakers and recorded.

### Fruit quality and netting grade evaluation

Destructive measurements of fruit length, fruit diameter, flesh thickness, soluble solid content (SSC), yield characteristics, and netting grade were conducted on 503 fruits, according to the research and analysis standards of the RDA of Korea^61^. Fruit shape index (FSI) was calculated as the ratio of fruit length to fruit diameter. SSC was measured by removing the peel, seeds, and placenta from the fruit, homogenizing the middle flesh using a blender, and analyzing the juice using a handheld refractometer (PAL-1; ATAGO Co., Ltd., Tokyo, Japan). The yield characteristics were determined by calculating the total yield per 10 acres using the average individual fruit weight after harvest.

This study employed trained human evaluators to assess and grade net quality more precisely. A group of eight evaluators, including senior researchers, researchers, and research assistants at the National Institute of Horticultural and Herbal Science, conducted blind evaluations on a five-point scale (1 = excellent, 2 = good, 3 = average, 4 = poor, and 5 = bad). The primary evaluation criteria during the visual assessment included net density, thickness, consistency of thickness, pattern uniformity, and proportion of the netted area, following the RDA guidelines.

### Water productivity (WP) and economic analysis

WP was calculated based on the melon yield ($\:Y$ and the amount ($\:{V}_{ir}$) of irrigation water applied, using Eq. 1^62^:

1 $$\:{WP}_{\:}=\frac{Y}{{V}_{ir}}$$

Economic analysis was conducted using the melon selling price, cultivation cost, and net profit. The selling price was determined based on the prevailing wholesale market price of the local agricultural products immediately after harvest. Cultivation costs included expenses for seeds, fertilizers, pesticides, labor, heating, and irrigation. The net profit was calculated by subtracting the total cultivation cost (substrate, fertilizer, and other fixed costs derived from the 2023 Agricultural Product Income Survey^11^) from the total sales revenue, which was estimated by multiplying the average market price by grade^63^ by the total production per 10a.

### Price adjustment by fruit quality grade

To align sugar content and net grade, evaluated as absolute and relative values, with market prices, normalization was performed on a 100-point scale. For sugar content, the normalization formula in Eq. 2 was used, whereas for net grade, the inverse normalization formula in Eq. 3 was applied, because lower values indicate superior quality.

2 $$\:{\begin{array}{c}Normalized\:score\\\:\left(Brix\right)\end{array}}_{\:}=\frac{Observed\:value-\:Minimum\:value}{Maximum\:value-\:Minimum\:value}\times\:100$$

3 $$\:{\begin{array}{c}Reverse\:normalized\\\:\:score\:\left(Net\right)\end{array}}_{\:}=\frac{Maximum\:value-\:Observed\:value}{Maximum\:value-\:Minimum\:value}\times\:100$$

The net melon prices by grade were compiled, as shown in Table 3, and converted to USD (exchange rate: 1 USD = 1,382 KRW) based on the average market price of muskmelons from the Seoul Agro-Fisheries & Food Corporation in May 2024^63^.

**Table 3:** Market price classification of netted melon by fruit grade, based on average wholesale prices from the Seoul Agro-Fisheries & food corporation as of May 2024. Prices are expressed in USD (exchange rate: 1 USD = 1,382 KRW). This price table was used to calculate normalized Brix and reverse-normalized net scores, and to estimate the final market grade and projected revenue in the economic analysis.

	Wholesale price (USD)			
Fruit grade	Mean	Max	Min	SD
Premium	7.38	10.42	4.64	1.73
Superior	6.65	10.07	3.87	1.91
Medium	5.29	8.69	2.38	1.87
Inferior	3.33	6.03	1.68	1.38

For premium market products targeting department stores and gift markets, external quality attributes such as netting are as important as sugar content for market value. In contrast, for public markets, such as direct-to-consumer sales and large retail stores, sugar content is the primary market value determinant because fruits are covered with protective mesh packaging. Therefore, for the premium market, the weighting factors for sugar content ($\:\alpha\:$) and netting ($\:\beta\:$) were set at 0.7 and 0.3, respectively, and 0.8 and 0.2, respectively, for the public market. Based on these weightings, the market grade of the fruits was estimated by multiplying the normalized scores for sugar content and netting, calculated using Eqs. 2 and 3, by the respective market weighting factors (Eq. 4).

4 $$\:{Estimated\:market\:grade}_{\:}=\alpha\:\times\:\left(Sugar\:content\right)+\beta\:\times\:\left(Netting\right)$$

### Multi-objective optimization and Pareto analysis

#### Performance indicator integration

To evaluate trade-offs among multiple cultivation objectives, six key performance indicators were integrated: quality score (normalized premium market value), total yield (kg/10a), irrigation water productivity (kg/m³), economic value (USD/10a), sugar content (Brix), and average fruit weight (kg). For a fair comparison across different measurement units, all indicators were transformed to a 0–100 scale using min-max normalization (Eq. 5).

5 $$\:{n}_{ij}=\left(\frac{{y}_{ij}-{y}_{j}^{min}}{{y}_{j}^{max}{-y}_{j}^{min}}\right)\times\:100$$

### Three-dimensional Pareto frontier analysis

An optimization problem was formulated to identify efficient treatment combinations that simultaneously maximized fruit quality, yield, and irrigation water productivity. The multiobjective function $\:F\left(x\right)$ for treatment $\:x$ is defined as (Eq. 6)^30^:

6 $$\:\mathrm{M}\mathrm{a}\mathrm{x}\mathrm{i}\mathrm{m}\mathrm{i}\mathrm{z}\mathrm{e}\:F\left(x\right)={\left[{f}_{1}\left(x\right),{f}_{2}\left(x\right),{f}_{3}\left(x\right)\right]}^{T}$$

where $\:{f}_{1}$, $\:{f}_{2}$ and $\:{f}_{3}$ are the normalized scores for quality, yield, and water productivity, respectively.

A treatment combination was considered Pareto optimal when it was not dominated by the other treatments. The Pareto frontier was calculated based on the dominance relationships. A solution $\:{x}_{a}$ is said to dominate solution $\:{x}_{b}$ (denoted as $\:{x}_{a}\succ\:{x}_{b}$) if and only if (Eq. 7):

7 $$\:\forall\:k\in\:\left\{\mathrm{1,2},3\right\},{f}_{k}\left({x}_{a}\right)\ge\:{f}_{k}\left({x}_{b}\right)\wedge\:\exists\:m\in\:\left\{\mathrm{1,2},3\right\},{f}_{m}\left({x}_{a}\right)>{f}_{m}\left({x}_{b}\right)$$

Consequently, the set of Pareto-optimal solutions $\:{P}^{*}$ is defined as the set of non-dominated solutions within the feasible design space $\:{\Omega\:}$:

8 $$\:{P}^{*}=\left\{{x}_{a}\in\:{\Omega\:}\:\left|\nexists\:{x}_{b}\right.\in\:{\Omega\:}\:\mathrm{s}\mathrm{u}\mathrm{c}\mathrm{h}\:\mathrm{t}\mathrm{h}\mathrm{a}\mathrm{t}\:{x}_{b}\succ\:{x}_{a}\right\}$$

### Comprehensive performance heatmap

A performance comparison matrix was generated to intuitively visualize the treatment rankings across all indicators. Six key indicators (quality score, total yield, irrigation water productivity, economic value, flesh thickness, and fruit weight) were normalized on a scale of 0–100 to enable a fair comparison. The heatmap employed a viridis color scheme, where yellow (100 points) represented the optimal performance and dark purple (0 points) represented the poorest performance within each indicator category. To enable quantitative comparison, each cell in the heatmap displays a normalized performance score. For correlation analysis, a Pearson correlation coefficient matrix was added as a subplot to simultaneously identify the relationships among the indicators. The correlation heatmap applies an upper triangular mask to eliminate redundant information, and expresses the correlation strength using a blue scheme.

### Experimental design and statistical analysis

The experiment was designed as a three-way factorial arrangement in a randomized complete block design (RCBD) with three replicates. The three factors investigated were: (1) Cultivar (three levels: ‘Dalgona,’ ‘Hero,’ ‘Kingstar’), (2) Substrate Volume (two levels: 10 L and 20 L), and (3) Planting Density (two levels: 3 and 4 plants/slab). The specific dimensions of the substrate and planting details are listed in Table 4. For treatment implementation, the row spacing was uniformly set at 150 cm. The planting density treatments corresponded to 2.2 and 3.0 plants/m² in the 10 L substrate volume, and 2.0 and 2.7 plants/m² in the 20 L substrate volume, respectively.

**Table 4:** Experimental design for hydroponic netted melon cultivation showing the combinations of substrate volume and planting density.

Substrate volume (L)^z^	Plants/slab	Planting distance (cm)	Plant density (plants/m²)	Projected of plants per 10 a^y^
10	3	30.0 × 150	2.2	2222
	4	22.5 × 150	3.0	2963
20	3	33.3 × 150	2.0	2000
	4	25.0 × 150	2.7	2667

z Dimensions of 10 L slab: 60 × 15 × 7.5 cm; 20 L slab: 100 × 20 × 10 cm.

y 10 a is equivalent to 1.00.

Statistical analyses were performed using Python 3.9 with the statistical libraries Pandas, numpy, scipy, matplotlib, and statsmodels. Prior to the analysis, the normality of the data distribution was verified using the Shapiro-Wilk test, and the homogeneity of variance was assessed using Levene’s test. The data were then subjected to three-way analysis of variance (ANOVA) to evaluate the main and interaction effects of the factors. Statistical significance of the effects was determined using the F-test. When the ANOVA indicated significant differences (*P* $\:<$ 0.05), mean separations were performed using Tukey’s Honest Significant Difference (HSD) test.

## Data Availability

Data supporting the findings of this study can be found in related cited articles and/or from the corresponding author upon reasonable request.
